# Major depressive episode among university students in Southern Brazil

**DOI:** 10.11606/s1518-8787.2020054001540

**Published:** 2020-01-23

**Authors:** Betina Daniele Flesch, Gbènankpon Mathias Houvèssou, Tiago Neuenfeld Munhoz, Anaclaudia Gastal Fassa

**Affiliations:** I Universidade Federal de PelotasFaculdade de MedicinaPrograma de Pós-graduação em EpidemiologiaPelotasRSBrasilUniversidade Federal de Pelotas. Faculdade de Medicina. Programa de Pós-graduação em Epidemiologia. Pelotas, RS, Brasil; II Universidade Federal de PelotasFaculdade de MedicinaPelotasRSBrasilUniversidade Federal de Pelotas. Faculdade de Medicina. Curso de graduação em Psicologia. Pelotas, RS, Brasil

**Keywords:** Students, psychology, Higher Education, Depression, epidemiology, Depressive Disorder, epidemiology, Risk Factors, Mental Health

## Abstract

**INTRODUCTION:**

Depression is the leading cause of disability around the world, and it has been increasingly affecting young people. This study evaluates the prevalence and factors associated with major depression in university students, with emphasis on the influence of the academic field, chosen study area and the environment they are inserted.

**METHODS:**

A census of students who entered the university in the first semester of 2017 was held at a university in Southern Brazil. The outcome of major depressive episode was evaluated using the Patient Health Questionnaire*-*9, considered when the individual had five or more depressive symptoms for at least one week. Its prevalence was estimated, and the associated factors were examined by the hierarchical multivariable analysis using the Poisson regression model.

**RESULTS:**

A total of 32% (95% confidence interval 29.9–34.2) of university students presented a major depressive episode, and the problem was more frequent among women (prevalence ratio [PR] = 1.59); people aged 21 to 23 years (PR = 1.24); those with a family history of depression (PR = 1.27); minorities’ sexual orientation (homosexuals, PR = 1.64, and bisexuals, PR = 1.69); who lived with friends or colleagues (PR = 1.36); students in the area of applied social and human sciences (PR = 1.28), and linguistics, language and literature, and art (PR = 1.25). The worst academic performance (PR = 2.61), alcohol abuse (PR = 1.25), and illicit drug use (PR = 1.30) were also positively associated with major depressive episode.

**CONCLUSION:**

In addition to individual, family, and behavioral aspects, already described as risk factors for major depressive episodes in the general population, academic aspects also influence the occurrence of depression among university students. Considering the high prevalence of major depressive episode and its negative impact on health, public and institutional policies are necessary to focus on students’ mental health promotion and care.

## INTRODUCTION

Depression is the main health problem and the leading cause of disability worldwide, reaching more than 300 million people and having increased 18% from 2005 to 2015^1^. According to the 2013 National Health Survey (NHS), the prevalence of self-reported depression diagnosis in adults was higher in Rio Grande do Sul (13.2%) than in Brazil (7.6%)^2^.

The literature also points out this problem among university students, showing that 15 to 25% of them develop some type of mental disorder during graduation, and depression is one of the most prevalent ones^3^. Both in Brazil and in other countries, when depression was evaluated in university students by the DSM-5 and the ICD-10, the prevalence varied between 5 and 15%^4,5^, and when it was tracked with questions about the depressive symptomatology, the results varied from 30 to 50%^6,7^.

As in the population studies, the prevalence of depression among university students is higher in women, older adults and people of low socioeconomic status. However, among university students, depression may be related to life stage transitions. It was positively associated with moving away from family and needing to adapt to the university environment^5^. In addition, academic requirements, financial difficulties, and concerns about the future can act as triggers for the depressive symptomatology^9,10^.

The major depressive episode (MDE) is characterized by the presence of depressed, empty or irritable mood, accompanied by somatic and cognitive changes that significantly affect the individual’s functional capacity for at least one week^11^. MDE leads individuals to isolationism, impacting daily activities, causing the reduction in physical activities, influencing appetite, either with gain or weight loss, and negatively affecting performance and academic productivity^12^. Besides, studies indicate that depression is associated with suicidal ideation and suicide risk^3,13^. However, despite the high prevalence of depression and its negative impact on the quality of life, part of the population does not recognize depressive symptoms as a disease, which slows its diagnosis and treatment, hindering suicide prevention^9^.

Studies that measure the occurrence of depression among university students use different measurement instruments and cutoff points, being sometimes restricted to some courses, which limits the extrapolation of the results to the general population of university students. Few studies detail the intensity and degrees of depression, as well as the main depressive symptoms^14^. In addition, studies address the main depression risk factors, which are common to the general population, without emphasizing specific aspects of university students.

This study evaluates the prevalence and factors associated with major depression in university students, with emphasis on the influence of the academic field, study area and the environment they are inserted.

## METHODS

The Graduate Program in Epidemiology of the Universidade Federal de Pelotas (UFPel) conducted a research consortium entitled *Saúde do Estudante Universitário *(SEU – University Student Health). As part of this consortium, a cross-sectional census study on undergraduate students was conducted in 80 UFPel in-person courses, with admission in the first semester of 2017 and with students still enrolled in the second half of 2017. Data were collected covering all masters’ dissertation themes of the program, thus optimizing human and financial resources^15^.

The number of students needed to evaluate the prevalence of major depressive episode was 1,328, considering the 30% prevalence estimate, a margin of error of two percentage points, the confidence level of 95%, and adding 10% for losses. For the association calculation, the largest sample size required was 1,291 to examine the association with the economic level. The following items were used as parameters: 80% statistical power, 95% confidence interval, exposed/unexposed ratio of 7:3, 15% prevalence of major depressive episode in non-exposed, and 1.5 relative risk, adding 10% for losses and 15% for the confounding factor control. The sample size calculation was performed in OpenEpi, version 3.01.

To meet the research objectives of all master’s students involved in the consortium, 2,706 students were included in the study, representing all eligible students. Undergraduates under the age of 18 years were excluded, as well as those who gave up or performed enrollment locks throughout the research period.

Among the independent variables, the considered demographic aspects were biological sex (male or female), age (in full years, categorized in 18, 19–20, 21–23 and ≥ 24 years old), skin color or ethnicity (white, black, mixed or other); the family history of self-reported depression (yes or no), considering family members “those with whom you have daily coexistence or blood tie,” and the predominant sexual orientation (heterosexual, bisexual, asexual or homosexual). Socioeconomic status was also investigated, classified according to the *Associação Brasileira de Empresas de Pesquisa* (ABEP – Brazilian Association of Research Companies)^16^ (A, B, C or D/E) and measured by the consumer goods, the region of origin (Pelotas, another city in Rio Grande do Sul, another state of Brazil, or another country), and with whom the university student lives (alone, parents or family, spouse, friends, or colleagues). In addition, the examined academic aspects were related to the area of knowledge of the course (exact and land/agrarian sciences and engineering, health and biological sciences, applied social and human or linguistic sciences, language and literature, and arts) and to the self-reported academic performance (bad/very bad, moderate, good or very good/excellent). The following behavioral variables were also evaluated: smoking (smoker, former smoker and non-smoker), harmful alcohol use according to the *Audit* considering the cutoff point ≥ 8 points (yes or no), and consumption of illicit substances in the last month (yes or no).

MDE was evaluated using the algorithm of the questionnaire Patient Health Questionnaire-9 (PHQ-9), which is a validated instrument in the population of Pelotas^17^, because it allows the diagnosis of MDE and its concise structure, and it is easy to understand. MDE was considered as the presence of five or more symptoms in the two weeks prior to the data collection, with at least one depressed mood or anhedonia, with each having occurred during “one week or more” or “almost every day,” except for symptom nine (“In the last two weeks, how many days did you think about hurting yourself or that it would be better to be dead?”), for which the occurrence was considered for “less than a week,” “a week or more” or “almost every day.” The prevalence of depression was also evaluated, according to severity levels, classified as without depression (1 to 4 points), mild depression (5 to 9), moderate depression (10 to 14), moderately severe depression (15 to 19), or severe depression (20 to 27 points)^18^.

A pilot study was conducted in October 2017 among students who entered the second semester of 2016, with self-applied questionnaire on paper, to evaluate and detect possible interpretation failures, the time required to answer the questionnaire, and the logistical aspects of the study.

The study data collection was performed between November 2017 and July 2018, by self-applied and anonymous questionnaire in tablets, using the Research Electronic Data Capture program (RedCap), or paper, when the many students exceeded the number of available tablets at the time or in case of the student’s preference. The masters’ students actively searched the students in the classroom after the consent of the coordinator and of the professor of the course. When they were not found in the classroom, due to absence or because they were not enrolled in that discipline, they were sought on another day, preferably in another course. To perform a good quality data collection, an application manual providing the possible doubts of the instrument was elaborated, the master’s students were trained to supervise the field work, weekly meetings were held aiming at the standardization of data collection, and partial data were analyzed to identify any early problems in the questionnaire.

Data were analyzed using the Stata program, version 12.0. First, a descriptive analysis of the outcome and the independent variables was performed to characterize the sample; subsequently, the prevalence of depression was estimated with the respective confidence interval of 95% (95%CI). A hierarchical multivariate analysis was conducted using the Poisson regression model and adjustment for robust variance. In the first level, demographic and socioeconomic variables were included; in the second level, the region of origin, with whom they live, and the academic aspects (the area of knowledge and the academic performance) were included; and in the third level, the behavioral variables.

The associations of the independent variables with the outcome were estimated by the prevalence ratios and their respective 95% confidence intervals, the heterogeneity chi-square test, and when possible, the linear trend test. In the multivariate analysis, aiming to adjust for confounding factors, the associated outcome variables with p-value < 0.20 were maintained in the model. The variables that presented a p-value < 0.05 were considered associated.

The project was approved by the Research Ethics Committee of the Faculdade de Medicina of UFPel, in October 2017 with no. 79250317.0.0000.5317. The theme of the study was explained to the students, and they were guaranteed the right to non-participation and confidentiality of the provided information. The questionnaire was only applied after the signing of the informed consent form.

## RESULTS

A total of 1,865 students were interviewed; 1,827 of them answered all PHQ-9 questions and were included in these analyses. Considering the percentage of eligible students who entirely answered the PHQ-9, the response rate was 67.5%. In the study population, 55% were women, 34.4% were between 19 and 20 years old, 72% self-declared whites, 56% reported a family history of depression, 44% were of the B economic level according to ABEP, 46% originated from Pelotas and 35% originated from other cities in the Rio Grande do Sul, 33% consumed alcohol and 23% took illicit substances in the last month. According to the algorithm for diagnosis of the PHQ-9, 32% (95%CI 29.9–34.2) presented MDE. Evaluating the depression severity levels^18^, 29.7% (95%CI 27.6–31.8) presented mild depression, 22.3% (95%CI 20.5–24.3) moderate depression, 15.2% (95%CI 13.6–16.9) moderately severe depression, and 15% severe depression (95%CI 13.4–17.7), according to Table 1.

**Table 1:** Description of the sample according to demographic, socioeconomic, and academic characteristics, and prevalence of major depressive episode (MDE) among university students. Pelotas (RS), Brazil, 2017 (n = 1827).

Variables	N (%)	% MDE	P-value
Sex			< 0,001
Male	820 (44,9)	23,3	
Female	1.005 (55,1)	39,1	
Age			0,019
18 years	398 (21,9)	27,9	
19–20 years	624 (34,4)	34,5	
21–23 years	422 (23,3)	35,8	
24 or older	371 (20,4)	28,3	
Skin Color/Ethnicity (self-reported)			0,075
White	1.311 (71,8)	30,7	
Black	240 (13,2)	39,2	
Mixed	243 (13,3)	32,1	
Other	31 (1,7)	35,5	
Family history of depression			< 0,001
No	794 (43,5)	26,2	
Yes	1.030 (56,5)	36,5	
Sexual orientation			< 0,001
Heterosexual	1.361 (74,9)	27,1	
Homosexual	143 (7,9)	42,0	
Bisexual	237 (13,0)	54,0	
Asexual	76 (4,2)	32,9	
Socioeconomic level			0,009a
A	261 (14,9)	28,4	
B	773 (44,3)	29,8	
C	634 (36,4)	34,4	
D/E	76 (4,4)	40,8	
Region of origin			0,204
Pelotas	831 (45,6)	30,3	
Another city in Rio Grande do Sul	638 (35,0)	31,7	
Another state	352 (19,3)	36,7	
Another country	3 (0,2)	33,3	
Who they live with			0,003
Alone	229 (12,6)	26,6	
Parents or family members	913 (50,1)	31,7	
Spouse	205 (11,3)	26,3	
Friends and colleagues	476 (26,1)	38,3	
Courses (large areas according to CNPq)			0,008
Exact and earth/agrarian sciences, and engineering	530 (29,0)	27,0	
Health and biological sciences	324 (17,7)	30,6	
Applied social and human sciences	632 (34,6)	34,3	
Linguistics, language and literature, and arts	341 (18,7)	37,0	
Academic performance			< 0,001
Terrible	25 (1,37)	84,0	
Very bad	78 (4,3)	56,4	
Moderate	605 (33,1)	36,7	
Good	729 (39,9)	27,7	
Very good	340 (18,6)	23,5	
Excellent	50 (2,7)	32,0	
Smoking			< 0,001
Non-smoker	1.338 (73,3)	28,3	
Former smoker	201 (11)	47,8	
Smoker	287 (15,7)	38,3	
Alcohol abuse^b^			< 0,001
No	1.118 (66,8)	27,7	
Yes	557 (33,3)	40,6	
Use of illicit substances (last month)			< 0,001
No	1.404 (76,9)	28,5	
Yes	423 (23,1)	43,7	
MDE diagnosis by PHQ-9			
No	1.242 (68,0)	65,9–70,2^c^	
Yes	585 (32,0)	29,8–34,1^c^	
Depression severity levels by the PHQ-9			
No depression	326 (17,8)	16,2–19,7^c^	
Mild depression	542 (29,7)	27,6–31,8^c^	
Moderate depression	408 (22,3)	20,5–24,3^c^	
Moderately severe depression	277 (15,2)	13,6–16,9^c^	
Severe depression	274 (15,0)	13,4–17,7^c^	

CNPq: *Conselho Nacional de Desenvolvimento Científico e Tecnológico*; PHQ-9: Patient Health Questionnaire-9; 95%CI: 95% confidence interval

^a^ P-value for trend.

^b^ For up to 152 individuals, information about at least one variable could not be obtained.

^c^ 95% confidence interval.

In the adjusted analysis, female students (prevalence ratio [PR] = 1.59; 95%CI 1.37–1.86), and bisexual students (PR = 1.69; 95%CI 1.44–1.98), and homosexuals (PR = 1.64; 95%CI 1.31–2.06) presented an average 60% risk for MDE. The students aged between 21 and 23 years had a 24% higher risk (95%CI 1.01–1.53) compared with the new students aged 18 years, and among those with a family history of depression, the MDE risk was 27% (95%CI 1.10–1.47), as shown in Table 2.

**Table 2:** Factors associated with major depressive episode among university students. Pelotas (RS), Brazil, 2017.

Variables	Crude analysis		Adjusted analysis	
	
	PR (95%CI)	P-value	PR (95%CI)	P-value
1^st^ level				

Sex		< 0,001		< 0,001
Male	Ref		Ref	
Female	1,68 (1,45–1,94)		1,59 (1,37–1,86)	
Age		0,021		0,068
18 years	Ref		Ref	
19 and 20 years	1,24 (1,02–1,50)		1,22 (1,00–1,48)	
21 to 23 years	1,28 (1,05–1,57)		1,24 (1,01–1,53)	
24 or older	1,02 (0,81–1,27)		1,03 (0,81–1,30)	
Skin Color/Ethnicity		0,026		0,063
White	Ref		Ref	
Mixed	1,06 (0,88–1,28)		1,13 (0,94–1,37)	
Black/other	1,28 (1,07–1,53)		1,23 (1,02–1,49)	
Family history of depression		< 0,001		0,008
No	Ref		Ref	
Yes	1,39 (1,21–1,61)		1,27 (1,10–1,47)	
Sexual orientation		< 0,001		< 0,001
Heterosexual	Ref		Ref	
Homosexual	1,55 (1,25–1,91)		1,64 (1,31–2,06)	
Bisexual	1,99 (1,72–2,31)		1,69 (1,44–1,98)	
Asexual	1,21 (0,87–1,69)		1,18 (0,85–1,63)	
Socioeconomic level		0,009^a^		0,081^a^
A	Ref		Ref	
B	1,05 (0,84–1,31)		1,05 (0,85–1,30)	
C	1,21 (0,97–1,51)		1,14 (0,92–1,42)	
D/E	1,44 (1,03–2,01)		1,32 (0,93–1,86)	

2^nd^ level				

Who they live with		0,003		0,037
Alone	Ref		Ref	
Parents or family members	1,19 (0,94–1,50)		1,23 (0,95–1,58)	
Spouse	0,99 (0,72–1,35)		1,00 (0,72–1,39)	
Friends and colleagues	1,43 (1,12–1,82)		1,36 (1,05–1,77)	
Courses (large areas according to CNPq)		0,009		0,038
Exact and earth/agrarian sciences, and engineering	Ref		Ref	
Health and biological sciences	1,13 (0,91–1,41)		1,10 (0,89–1,36)	
Applied social and human sciences	1,27 (1,07–1,52)		1,28 (1,07–1,53)	
Linguistics, language and literature, and arts	1,37 (1,12–1,67)		1,25 (1,01–1,54)	
Academic performance		< 0,001		< 0,001
Excellent/very good	Ref		Ref	
Good	1,13 (0,91–1,39)		1,15 (0,89–1,48)	
Moderate	1,49 (1,22–1,83)		1,51 (1,18–1,95)	
Poor/terrible	2,56 (2,04–3,22)		2,61 (1,87–3,64)	

3^rd^ level				

Alcohol abuse		< 0,001		0,003
No	Ref		Ref	
Yes	1,46 (1,27–1,68)		1,25 (1,08–1,45)	
Use of illicit substances (last month)		< 0,001		0,001
No	Ref		Ref	
Yes	1,53 (1,34–1,76)		1,30 (1,11–1,52)	

PR: prevalence ratio; 95%CI: 95% confidence interval; CNPq: *Conselho Nacional de Desenvolvimento Científico e Tecnológico*.

^a^ P-value for trend.

Students who reported living with friends and colleagues had a 36% MDE higher risk (95%CI 1.05–1.77) compared with those who lived alone. Students of social and human science courses (95%CI 1.07–1.53) and those of linguistics, language and literature, and art (95%CI 1.01–1.54) presented an average 25% MDE higher risk when compared with those of the courses of exact and land/agrarian sciences, and engineering. The academic performance was inversely associated with the MDE occurrence, being 2.6 times higher (95%CI 1.87–3.64) among students who reported “very poor/poor” performance when compared with those who had “excellent/very good” performance. The alcohol abuse (PR = 1.25; 95%CI 1.08–1.45) and the use of illicit substances in the last 30 days (PR = 1.30; 95%CI 1.11–1.52) were also MDE risk factors (Table 2).

## DISCUSSION

Among the surveyed university students, 32% had MDE and the problem was more frequent among women, aged 21 to 23 years, with a family history of depression, with minorities’ sexual orientation (homosexuals and bisexuals), who lived with friends and colleagues, and students in the area of applied social and human sciences, as well as linguistics, language and literature, and arts. The worst academic performance, the alcohol abuse, and the illicit drug use were also positively associated with MDE.

Most studies on Brazilian university students used convenience samples and were limited to specific courses, especially to the health area^14,19^. The most used instrument to measure the prevalence of depression symptoms was the Beck Depression Inventory (BDI). A survey that evaluated 1,039 students from health courses in a southern Brazilian city indicated a prevalence of mild, moderate, or severe depressive symptomatology in 28.3% of the students^22^. Among the studies that investigated the diagnosis of depression, De Melo Cavestro and Rocha^23^ evaluated 342 university students of health courses using the Mini International Neuropsychiatric Interview (Mini) and observed the 10.5% MDE prevalence^3^. Vasconcelos et al.^23^ evaluated a convenience sample of 234 medical students through the Hospital Anxiety and Depression Scale (HADS) and found suggestive symptoms of the disorder at 5.6%.

No studies using the PHQ-9 among university students in Brazil were found. In this study, the prevalence of depression was higher than among university students in other countries that used the PHQ-9. In Australia, it was 7.9%^5^. In England, the prevalence of severe depression was 5.6% for medical students and 12.7% for other courses, while moderate depression was 10.8% among medical students and 17.7% in other courses^24^. The study by Leppink et al.^25^, conducted in the United States using the PHQ-9, found a 37.7% prevalence for mild to moderate depressive symptoms and 4.4% for severe depressive symptoms, also lower than that found in this study.

The 50% higher risk of MDE among women is consistent with the literature, which points to a large health disparity between sexes, reaching a peak in adolescence^4,5,13,14,26,27^. The sex gap may be directly related to vulnerability, prejudice, and sex discrimination, and because women report and better recognize depressive symptoms seeking more help for health problems than men, as well as because they often face multiple journeys, taking care of their families, work, and study^28^.

The highest prevalence of MDE among university students was found in the range of 21 to 23 years of age, similar to that observed by other authors who detected the highest prevalence of depression among older students, aged 20 years or older^5,8^. Olsson and Von Knorring^29^ observed that 25% of adults with depressive disorders report having their first depressive episode before the age of 18 years.

The positive association between the presence of family history of depression and MDE is in accordance with the literature^30,31^. While some studies claim that the relationship between family history and depression is genetic, others consider that it is more related to the family life^32^. Bahls et al.^33^ pointed out in their systematic review that the family history of depression is the main risk factor for its development among children and adolescents, increasing it by up to three times. Homosexuality and bisexuality, sexual orientation of minorities, were risk factors for MDE in this study, agreeing with the literature findings^25,34^. The higher occurrence of depression in the population with minority sexual orientation may be related to prejudice, lack of acceptance, and stigmatization imposed by the society, as well as to the family distancing and lower support network^34^.

This study found the highest prevalence of MDE among students who reported living with friends or colleagues, which may be related to the distance of their families and support network, as well as the need to adapt to the routine of shared republics, pensions and student housing^7,23^.

Although most studies found in the literature specifically deal with university students from health courses, our study observed the highest MDE prevalence among students in the areas of humanities, arts and linguistics, a result similar to another Brazilian study^14^. The higher prevalence of depression in humanities students may be related to particular academic requirements and routines, and/or students’ greater difficulty to face them^14^. Previous studies have observed that the course dissatisfaction of the students was also positively related to the MDE and MDD (major depressive disorder) occurrence^27,30^.

A negative relationship was observed between academic performance and MDE, as already described in the literature. The good academic performance is a significant marker of the students’ success and development, indicating a positive response to competitiveness and to the high level of demand in the university. This association is subjected to a two-way direction, since the already depressed student tends to find greater difficulties in performing their daily activities, as well as being depressed by the poor performance achieved. In addition, students with MDE can make a more critical and pessimistic self-assessment^4,7,12,27^.

The positive association between illicit drug use, alcohol, and depression is consistent with the literature findings^23,26^. The causal relationship between these outcomes is not fully enlightened, and it is described that neurophysiological and metabolic alterations resulting from alcohol exposure may be related to the depression development^35^. Drug use may be related to MDE as both a risk factor for the development of biochemical and neurophysiological alterations, as well as because it is a marker of the student’s vulnerability. The literature reports that illicit drug use is related to suicidal thoughts, sadness, loneliness, and difficulty sleeping, and that the presence of alcohol abuse doubles the chance of depressive disorder occurrence, being the opposite also true^35,36^. Thus, considering this cross-sectional study, this association may be affected by reverse causality and/or two-way direction.

This study found no association between the economic level and skin color with MDE, although these associations are widely reported in the literature. The existing quota policy in the institution seeks to expand the number of students from public schools, blacks, and aborigines. Although quota students come from the most vulnerable groups, the arduous selection process makes them the most privileged among their peers, those most proactive or better able to face adversity, which may explain the absence of the association with MDE^4,5,31^.

The change of school semester in the data collection period and the course withdrawal or abandonment by students implied a considerable loss, more concentrated in men and older individuals, for courses in exact and land/agrarian sciences and engineering, which can cause inaccuracies in the prevalence estimation. Although this study advances in relation to the literature by evaluating all courses, the external findings validity is limited because it refers to a specific university.

As positive aspects, the high descriptive and screening value for the mental health of students stands out, which will serve as a basis for reception and for interventions to be performed in the university population, and the self-applying method of the instrument enables students to address sensitive issues, such as MDE and its symptoms. The fact the PHQ-9 was not used in other studies in Brazilian university students limits the comparability of the findings; however, as it was recently validated for the Brazilian population and has the advantage of providing a diagnosis of MDE, it is desirable for future studies to start using this instrument^17^.

In addition to individual, family, and behavioral aspects, similar to those already described as risk factors for MDE in the general population, academic aspects also influence the occurrence of depression among university students. Considering the high prevalence of MDE and its negative impact on health, quality of life, and academic development of university students, public and institutional policies are needed to promote health, especially mental health, as well as a structure to meet the students’ demands for adequate healthcare.
