# Supplementary material for: Major depressive episode among university students in Southern Brazil
Source: Rev Saude Publica. 2020 Jan 23;54:11. doi: 10.11606/s1518-8787.2020054001540 (PMC6986868; doi:10.11606/s1518-8787.2020054001540)
Supplement: Translation

# Translation: Episódio depressivo maior entre universitários do sul do Brasil

**Authors:** Betina Daniele Flesch, Gbènankpon Mathias Houvèssou, Tiago Neuenfeld Munhoz, Anaclaudia Gastal Fassa

**Affiliations:** I Brasil Universidade Federal de Pelotas. Faculdade de Medicina. Programa de Pós-graduação em Epidemiologia. Pelotas, RS, Brasil; II BrasilUniversidade Federal de Pelotas. Faculdade de Medicina. Curso de graduação em Psicologia. Pelotas, RS, Brasil

**Keywords:** Estudantes, psicologia, Educação Superior, Depressão, epidemiologia, Transtorno Depressivo, epidemiologia, Fatores de Risco, Saúde Mental

## Abstract

**INTRODUÇÃO:**

A depressão é a principal causa de incapacidade em todo o mundo, atingindo cada vez mais os jovens. Este estudo avalia a prevalência e fatores associados ao episódio depressivo maior em universitários, com ênfase na influência do meio acadêmico, área de estudo escolhida pelo universitário e ambiente onde ele está inserido.

**METODOLOGIA:**

Realizou-se um censo dos universitários ingressantes do primeiro semestre de 2017 em uma universidade do sul do Brasil. O desfecho episódio depressivo maior foi avaliado a partir do questionário *Patient Health Questionnaire-9*, considerado quando o indivíduo apresentava cinco ou mais sintomas depressivos por pelo menos uma semana. Sua prevalência foi estimada e os fatores associados foram examinados pela análise multivariável hierarquizada utilizando a regressão de Poisson com seleção para trás.

**RESULTADOS:**

Um total de 32% (intervalo de confiança de 95% 29,9–34,2) dos universitários apresentou episódio depressivo maior, e o problema foi mais frequente entre indivíduos do sexo feminino (razão de prevalências [RP] = 1,59), de 21 a 23 anos de idade (RP = 1,24), com histórico familiar de depressão (RP = 1,27), com orientação sexual de minorias (homossexuais, RP = 1,64, e bissexuais, RP = 1,69), que moravam com amigos ou colegas (RP = 1,36), estudantes da área das ciências sociais aplicadas e humanas (RP = 1,28) e de linguística, letras e artes (RP = 1,25). O pior desempenho acadêmico (RP = 2,61), o uso abusivo de álcool (RP = 1,25) e o consumo de drogas ilícitas (RP = 1,30) também estiveram positivamente associados ao episódio depressivo maior.

**CONCLUSÃO:**

Além dos aspectos individuais, familiares e comportamentais, semelhantes aos já descritos como fatores de risco para episódio depressivo maior na população em geral, aspectos acadêmicos também influenciam a ocorrência de depressão entre universitários. Considerando a alta prevalência de episódio depressivo maior e seu impacto negativo na saúde, são necessárias políticas públicas e institucionais que enfoquem a promoção da saúde e atenção à demanda de saúde mental dos estudantes.

## INTRODUÇÃO

A depressão é o principal problema de saúde e principal causa de incapacidade em todo o mundo, atingindo mais de 300 milhões de pessoas e apresentando um aumento de 18% entre 2005 e 2015^1^. Segundo a Pesquisa Nacional de Saúde (PNS) de 2013, a prevalência de diagnóstico de depressão autorrelatado em adultos foi maior no Rio Grande do Sul (13,2%) do que no Brasil (7,6%)^2^.

A literatura aponta que esse problema também é importante entre universitários, mostrando que 15 a 25% deles desenvolvem algum tipo de transtorno mental durante a graduação, sendo a depressão um dos mais prevalentes^3^. Tanto no Brasil quanto em outros países, quando a depressão em universitários foi avaliada pelo DSM-5 e CID-10, as prevalências variaram entre 5 e 15%^4,5^, e quando rastreada através de perguntas sobre a sintomatologia depressiva, se apresentaram entre 30 e 50%^6,7^.

Assim como em estudos populacionais, a prevalência de depressão entre universitários é maior em indivíduos do sexo feminino, mais velhos e de nível econômico mais baixo. Entretanto, em universitários, a depressão pode estar relacionada a aspectos relativos à transição nessa etapa da vida. Ela foi positivamente associada à saída da casa dos pais, com consequente afastamento da família e necessidade de adaptação ao ambiente da universidade^5^. Além disso, considera-se que as exigências acadêmicas, dificuldades financeiras e preocupações com o futuro podem atuar como gatilhos no desencadeamento de sintomatologia depressiva^9,10^.

O episódio depressivo maior (EDM) se caracteriza pela presença de humor deprimido, vazio ou irritável, acompanhado de mudanças somáticas e cognitivas que afetam significativamente a capacidade funcional do indivíduo por pelo menos uma semana^11^. O EDM leva os indivíduos ao isolacionismo, impactando as atividades diárias, provocando a redução da prática de atividades físicas, influenciando no apetite, seja com o ganho ou com a perda de peso, e afetando negativamente o desempenho e a produtividade acadêmica^12^. Além disso, estudos apontam que a depressão está associada à ideação suicida e ao risco de suicídio^3,13^. Entretanto, apesar da alta prevalência de depressão e de seu impacto negativo na qualidade de vida, boa parte da população não reconhece os sintomas depressivos como doença, o que retarda o diagnóstico e o tratamento, dificultando a prevenção do suicídio^9^.

Os estudos que mensuram a ocorrência de depressão entre os universitários utilizam diferentes instrumentos de medida e pontos de corte e, às vezes, são restritos a alguns cursos, o que limita a extrapolação dos resultados para a população de universitários. Poucos estudos detalham a intensidade e os graus de depressão encontrados, bem como os principais sintomas depressivos^14^. Além disso, os estudos abordam os principais fatores de risco para depressão, que são comuns à população em geral, sem enfatizar aspectos específicos dos universitários.

Este estudo avaliou a prevalência e os fatores associados ao episódio depressivo maior em universitários, com ênfase na influência do meio acadêmico, da área de estudo escolhida pelo universitário e do ambiente onde ele está inserido.

## MÉTODOS

O Programa de Pós-graduação em Epidemiologia da Universidade Federal de Pelotas (UFPel) realizou um consórcio de pesquisas intitulado Saúde do Estudante Universitário (SEU). Como parte desse consórcio, foi realizado um estudo transversal censitário dos alunos de graduação, dos 80 cursos presenciais da UFPel, com ingresso no primeiro semestre de 2017 e que estavam matriculados no segundo semestre de 2017. Foram coletados dados que abrangiam os temas das dissertações de todos os mestrandos do programa, otimizando assim recursos humanos e financeiros^15^.

O número de estudantes necessários para avaliar a prevalência de episódio depressivo maior foi de 1.328, considerando a estimativa de prevalência de 30%, uma margem de erro de dois pontos percentuais, o nível de confiança de 95% e acrescentando 10% para perdas. Para cálculo de associação, o maior tamanho de amostra necessário foi de 1.291 para examinar a associação com o nível econômico. Utilizaram-se como parâmetros o poder estatístico de 80%, nível de confiança de 95%, relação exposto/não exposto de 7:3, prevalência de episódio depressivo maior em não expostos de 15% e risco relativo de 1,5, acrescentando 10% para perdas e 15% para controle de fator de confusão. O cálculo de tamanho de amostra foi realizado no OpenEpi versão 3.01.

Para atender aos objetivos de pesquisa de todos os mestrandos envolvidos no consórcio, incluíram-se no estudo 2.706 estudantes, o que representou a totalidade dos estudantes elegíveis. Foram excluídos estudantes menores de 18 anos, bem como aqueles que desistiram ou realizaram trancamento de matrícula ao longo do período da pesquisa.

Entre as variáveis independentes, avaliaram-se os aspectos demográficos sexo biológico (masculino ou feminino), idade (em anos completos, categorizada em 18, 19–20, 21–23 e ≥ 24 anos), cor da pele ou raça (branca, preta, parda ou outra); o histórico familiar de depressão autorreferido (sim ou não), considerando familiares “aqueles com que você tem convivência cotidiana ou laço de sangue” e a orientação sexual predominante (heterossexual, bissexual, assexual ou homossexual). Também foram investigados o nível socioeconômico, classificado de acordo com a Associação Brasileira de Empresas de Pesquisa (ABEP)^16^ (A, B, C ou D/E) e medido através dos bens de consumo, a região de origem (Pelotas, outra cidade do Rio Grande do Sul, outro estado do Brasil ou outro país) e com quem o universitário mora (sozinho, pais ou familiares, cônjuge, amigos ou colegas). Além disso, foram examinados os aspectos acadêmicos relativos à área de conhecimento do curso (ciências exatas e da terra/agrárias e engenharias, ciências da saúde e biológicas, ciências sociais aplicadas e humanas ou linguística, letras e artes) e o desempenho acadêmico autorreferido (péssimo/muito ruim, razoável, bom ou muito bom/excelente). As variáveis comportamentais tabagismo (fumante, ex-fumante e não fumante), uso prejudicial de álcool segundo o *Audit* considerando o ponto de corte ≥ 8 pontos (sim ou não) e consumo de substâncias ilícitas no último mês (sim ou não) também foram avaliadas.

O EDM foi avaliado utilizando o algoritmo do questionário *Patient Health Questionnaire-9* (PHQ-9), que foi escolhido por ser um instrumento validado na população de Pelotas^17^, por possibilitar o diagnóstico do EDM e pela sua estrutura concisa e de fácil compreensão. Considerou-se EDM a presença de cinco ou mais sintomas nas duas semanas anteriores à coleta de dados, sendo pelo menos um o humor deprimido ou anedonia, com cada um tendo ocorrido durante “uma semana ou mais” ou “quase todos os dias”, exceto pelo sintoma nove (“Nas últimas duas semanas, quantos dias o(a) sr.(a) pensou em se ferir de alguma maneira ou que seria melhor estar morto(a)?”), para o qual foi considerada a ocorrência por “menos de uma semana”, “uma semana ou mais” ou “quase todos os dias”. Avaliou-se ainda a prevalência de depressão segundo os níveis de gravidade, classificada em sem depressão (1 a 4 pontos), depressão leve (5 a 9), depressão moderada (10 a 14), depressão moderadamente grave (15 a 19) ou depressão grave (20 a 27 pontos)^18^.

Para avaliar e detectar possíveis falhas de interpretação, o tempo necessário para responder ao questionário e aspectos logísticos do estudo, em outubro de 2017 foi realizado um estudo-piloto entre estudantes que ingressaram no segundo semestre de 2016, com questionário autoaplicado em papel.

A coleta de dados do estudo foi realizada entre novembro de 2017 e julho de 2018, por questionário autoaplicado e anônimo em *tablets*, utilizando o programa *Research Electronic Data Capture* (RedCap), ou papel, quando o número de alunos ultrapassava o número de *tablets* disponíveis no momento ou em caso de preferência do aluno. Os mestrandos fizeram busca ativa dos alunos em sala de aula após consentimento do coordenador do curso e do professor regente da disciplina em questão. Quando não eram encontrados em sala de aula, por ausência ou por não estarem matriculados naquela disciplina, foram buscados em outro dia, preferencialmente em outra disciplina do curso. Para realizar uma coleta de dados de boa qualidade, foi elaborado um manual de aplicação do instrumento prevendo as possíveis dúvidas, os mestrandos foram treinados para supervisionar o trabalho de campo, foram realizadas reuniões semanais visando à padronização da coleta de dados e os dados parciais foram analisados quanto à sua consistência para identificar precocemente eventuais problemas no questionário.

Os dados foram analisados no programa Stata 12.0. Primeiramente foi realizada uma análise descritiva do desfecho e das variáveis independentes para caracterização da amostra; posteriormente foi calculada a prevalência de depressão com o respectivo intervalo de confiança de 95% (IC95%). Uma análise multivariável hierarquizada foi conduzida utilizando a regressão de Poisson com seleção para trás e ajuste para variância robusta. No primeiro nível foram incluídas as variáveis demográficas e socioeconômicas; no segundo nível, a região de origem, com quem mora e os aspectos acadêmicos (área de conhecimento do curso e desempenho acadêmico); e no terceiro nível as variáveis comportamentais.

As associações das variáveis independentes com o desfecho foram calculadas através das razões de prevalências e seus respectivos intervalos de confiança de 95%, do teste do qui-quadrado de heterogeneidade e, quando possível, do teste de tendência linear. Na análise multivariável, objetivando ajuste para fatores de confusão, foram mantidas no modelo as variáveis associadas ao desfecho com valor-p < 0,20. Foram consideradas associadas as variáveis que apresentaram valor-p < 0,05.

O projeto foi aprovado pelo Comitê de Ética em Pesquisa da Faculdade de Medicina da UFPel, com o parecer nº 79250317.0.0000.5317 de outubro de 2017. Foi explicado aos alunos o tema da pesquisa, tendo sido garantido o direito à não participação e também a confidencialidade das informações prestadas. O questionário foi aplicado somente após a assinatura do termo de consentimento livre e esclarecido.

## RESULTADOS

Foram entrevistados 1.865 estudantes; 1.827 deles responderam a todas as questões do PHQ-9 e foram incluídos nestas análises. Considerando o percentual de alunos elegíveis que responderam o PHQ-9 em sua totalidade, a taxa de resposta foi de 67,5%. Na população estudada 55% eram do sexo feminino, 34,4% tinham idade entre 19 e 20 anos, 72% se autodeclaravam brancos, 56% relataram histórico familiar de depressão, 44% eram do nível econômico B segundo a ABEP, 46% eram originários de Pelotas e 35% de outras cidades do Rio Grande do Sul, 33% faziam uso abusivo de álcool e 23% fizeram uso de substâncias ilícitas no último mês. Segundo o algoritmo para diagnóstico do PHQ-9, 32% (IC95% 29,9–34,2) apresentaram EDM. Quando avaliados os níveis de gravidade da depressão^18^, 29,7% (IC95% 27,6–31,8) apresentaram depressão leve, 22,3% (IC95% 20,5–24,3) moderada, 15,2% (IC95% 13,6–16,9) moderadamente grave e 15% grave (IC95% 13,4–17,7), conforme a Tabela 1.

**Tabela 1:** Descrição da amostra segundo características demográficas, socioeconômicas e acadêmicas e prevalência de episódio depressivo maior (EDM) entre universitários. Pelotas (RS), Brasil, 2017 (n = 1827).

Variáveis	N (%)	% EDM	Valor-p
Sexo			< 0,001
Masculino	820 (44,9)	23,3	
Feminino	1.005 (55,1)	39,1	
Idade			0,019
18 anos	398 (21,9)	27,9	
19–20 anos	624 (34,4)	34,5	
21–23 anos	422 (23,3)	35,8	
24 ou mais	371 (20,4)	28,3	
Cor da pele/raça (autorreferida)			0,075
Branca	1.311 (71,8)	30,7	
Preta	240 (13,2)	39,2	
Parda	243 (13,3)	32,1	
Outra	31 (1,7)	35,5	
Histórico familiar de depressão			< 0,001
Não	794 (43,5)	26,2	
Sim	1.030 (56,5)	36,5	
Orientação sexual			< 0,001
Heterossexual	1.361 (74,9)	27,1	
Homossexual	143 (7,9)	42,0	
Bissexual	237 (13,0)	54,0	
Assexual	76 (4,2)	32,9	
Nível socioeconômico			0,009^a^
A	261 (14,9)	28,4	
B	773 (44,3)	29,8	
C	634 (36,4)	34,4	
D/E	76 (4,4)	40,8	
Região de origem			0,204
Pelotas	831 (45,6)	30,3	
Outra cidade do Rio Grande do Sul	638 (35,0)	31,7	
Outro estado	352 (19,3)	36,7	
Outro país	3 (0,2)	33,3	
Com quem mora			0,003
Sozinho(a)	229 (12,6)	26,6	
Pais ou familiares	913 (50,1)	31,7	
Cônjuge	205 (11,3)	26,3	
Amigos e colegas	476 (26,1)	38,3	
Cursos (grandes áreas segundo o CNPq)			0,008
Ciências exatas e da terra/agrárias e engenharias	530 (29,0)	27,0	
Ciências da saúde e biológicas	324 (17,7)	30,6	
Ciências sociais aplicadas e humanas	632 (34,6)	34,3	
Linguística, letras e artes	341 (18,7)	37,0	
Desempenho acadêmico			< 0,001
Péssimo	25 (1,37)	84,0	
Muito ruim	78 (4,3)	56,4	
Razoável	605 (33,1)	36,7	
Bom	729 (39,9)	27,7	
Muito bom	340 (18,6)	23,5	
Excelente	50 (2,7)	32,0	
Tabagismo			< 0,001
Não fumante	1.338 (73,3)	28,3	
Ex-fumante	201 (11)	47,8	
Fumante	287 (15,7)	38,3	
Uso abusivo de álcool^b^			< 0,001
Não	1.118 (66,8)	27,7	
Sim	557 (33,3)	40,6	
Uso de substâncias ilícitas (no último mês)			< 0,001
Não	1.404 (76,9)	28,5	
Sim	423 (23,1)	43,7	
Diagnóstico de EDM pelo PHQ-9			
Não	1.242 (68,0)	65,9–70,2^c^	
Sim	585 (32,0)	29,8–34,1^c^	
Níveis de gravidade de depressão pelo PHQ-9			
Sem depressão	326 (17,8)	16,2–19,7^c^	
Depressão leve	542 (29,7)	27,6–31,8^c^	
Depressão moderada	408 (22,3)	20,5–24,3^c^	
Depressão moderadamente grave	277 (15,2)	13,6–16,9^c^	
Depressão grave	274 (15,0)	13,4–17,7^c^	

CNPq: Conselho Nacional de Desenvolvimento Científico e Tecnológico; PHQ-9: *Patient Health Questionnaire-9*; IC95%: intervalo de confiança de 95%

^a^ Valor-p de tendência.

^b^ Para até 152 indivíduos não foi possível obter informações sobre pelo menos uma variável.

^c^ Intervalo de confiança de 95%.

Na análise ajustada, estudantes do sexo feminino (razão de prevalências [RP] = 1,59; IC95% 1,37–1,86) e estudantes bissexuais (RP = 1,69; IC95% 1,44–1,98) e homossexuais (RP = 1,64; IC95% 1,31–2,06) apresentaram um risco de cerca de 60% para EDM. Os alunos com idade entre 21 e 23 anos apresentaram 24% de risco (IC95% 1,01–1,53) em comparação aos alunos ingressantes de 18 anos e, entre aqueles que tinham histórico familiar de depressão, o risco de EDM foi de 27% (IC95% 1,10–1,47), como mostrado na Tabela 2.

**Tabela 2:** Fatores associados ao episódio depressivo maior entre universitários. Pelotas (RS), Brasil, 2017

Variáveis	Análise bruta		Análise ajustada	
	
	RP (IC95%)	Valor-p	RP (IC95%)	Valor-p
1º nível				

Sexo		< 0,001		< 0,001
Masculino	Ref		Ref	
Feminino	1,68 (1,45–1,94)		1,59 (1,37–1,86)	
Idade		0,021		0,068
18 anos	Ref		Ref	
19 e 20 anos	1,24 (1,02–1,50)		1,22 (1,00–1,48)	
21 a 23 anos	1,28 (1,05–1,57)		1,24 (1,01–1,53)	
24 ou mais anos	1,02 (0,81–1,27)		1,03 (0,81–1,30)	
Cor da pele/raça		0,026		0,063
Branca	Ref		Ref	
Parda	1,06 (0,88–1,28)		1,13 (0,94–1,37)	
Preta/outra	1,28 (1,07–1,53)		1,23 (1,02–1,49)	
Histórico familiar de depressão		< 0,001		0,008
Não	Ref		Ref	
Sim	1,39 (1,21–1,61)		1,27 (1,10–1,47)	
Orientação sexual		< 0,001		< 0,001
Heterossexual	Ref		Ref	
Homossexual	1,55 (1,25–1,91)		1,64 (1,31–2,06)	
Bissexual	1,99 (1,72–2,31)		1,69 (1,44–1,98)	
Assexual	1,21 (0,87–1,69)		1,18 (0,85–1,63)	
Nível socioeconômico		0,009^a^		0,081^a^
A	Ref		Ref	
B	1,05 (0,84–1,31)		1,05 (0,85–1,30)	
C	1,21 (0,97–1,51)		1,14 (0,92–1,42)	
D/E	1,44 (1,03–2,01)		1,32 (0,93–1,86)	

2º nível				

Com quem mora		0,003		0,037
Sozinho(a)	Ref		Ref	
Pais ou familiares	1,19 (0,94–1,50)		1,23 (0,95–1,58)	
Cônjuge	0,99 (0,72–1,35)		1,00 (0,72–1,39)	
Amigos e colegas	1,43 (1,12–1,82)		1,36 (1,05–1,77)	
Cursos (grandes áreas do CNPq)		0,009		0,038
Ciências exatas e da terra/agrárias e engenharias	Ref		Ref	
Ciências da saúde e biológicas	1,13 (0,91–1,41)		1,10 (0,89–1,36)	
Ciências sociais aplicadas e humanas	1,27 (1,07–1,52)		1,28 (1,07–1,53)	
Linguística, letras e artes	1,37 (1,12–1,67)		1,25 (1,01–1,54)	
Desempenho acadêmico		< 0,001		< 0,001
Excelente/muito bom	Ref		Ref	
Bom	1,13 (0,91–1,39)		1,15 (0,89–1,48)	
Razoável	1,49 (1,22–1,83)		1,51 (1,18–1,95)	
Muito ruim/péssimo	2,56 (2,04–3,22)		2,61 (1,87–3,64)	

3° nível				

Uso abusivo de álcool		< 0,001		0,003
Não	Ref		Ref	
Sim	1,46 (1,27–1,68)		1,25 (1,08–1,45)	
Uso desubstâncias ilícitas (no último mês)		< 0,001		0,001
Não	Ref		Ref	
Sim	1,53 (1,34–1,76)		1,30 (1,11–1,52)	

RP: razão de prevalências; IC95%: intervalo de confiança de 95%; CNPq: Conselho Nacional de Desenvolvimento Científico e Tecnológico

^a^ Valor-p de tendência.

Os alunos que relataram morar com amigos e colegas tiveram um risco de 36% (IC95% 1,05–1,77) para o EDM em comparação aos que moravam sozinhos. Estudantes de cursos de ciências sociais e humanas (IC95% 1,07–1,53) e aqueles de linguística, letras e artes (IC95% 1,01–1,54) apresentaram um risco de cerca de 25% para EDM quando comparados aos dos cursos de ciências exatas e da terra/agrárias e engenharias. O desempenho acadêmico esteve inversamente associado à ocorrência de EDM, sendo 2,6 vezes maior (IC95% 1,87–3,64) entre os estudantes que relatavam ter desempenho “muito ruim/péssimo” quando comparado aos que tinham desempenho “excelente/muito bom”. O uso abusivo de álcool (RP = 1,25; IC95% 1,08–1,45) e o uso de substâncias ilícitas nos últimos 30 dias (RP = 1,30; IC95% 1,11–1,52) também foram fatores de risco para o EDM (Tabela 2).

## DISCUSSÃO

Entre os universitários pesquisados, 32% apresentaram EDM e o problema foi mais frequente entre indivíduos do sexo feminino, de 21 a 23 anos de idade, com histórico familiar de depressão, com orientação sexual de minorias (homossexuais e bissexuais), que moravam com amigos e colegas e eram estudantes da área das ciências sociais aplicadas e humanas e da área de linguística, letras e artes. O pior desempenho acadêmico, o uso abusivo de álcool e o consumo de drogas ilícitas também estiveram positivamente associados ao EDM.

A maior parte dos estudos em universitários brasileiros utilizou amostras de conveniência e foi limitada a cursos específicos, sobretudo da área da saúde^14,19-21^. O instrumento mais usado para medir a prevalência de sintomas de depressão foi o Inventário de Depressão de Beck (BDI). Uma pesquisa que avaliou 1.039 estudantes de cursos da área da saúde de uma cidade do sul do Brasil apontou uma prevalência de sintomatologia depressiva leve, moderada ou grave em 28,3% dos alunos^22^. Entre os estudos que investigaram diagnóstico de depressão, De Melo Cavestro e Rocha^23^ avaliaram 342 universitários de cursos da área da saúde utilizando o *Mini International Neuropsychiatric Interview* (Mini) e observaram a prevalência de EDM de 10,5%^3^. Vasconcelos et al.^23^ avaliaram uma amostra de conveniência de 234 estudantes de medicina através da Escala Hospitalar de Ansiedade e Depressão (EHAD) e encontraram sintomas sugestivos do transtorno em 5,6%.

Não foram encontrados estudos utilizando o PHQ-9 entre universitários no Brasil. Neste estudo a prevalência de depressão foi superior à encontrada entre os universitários em outros países que utilizaram o PHQ-9. Na Austrália, ela foi de 7,9%^5^. Na Inglaterra a prevalência de depressão severa foi de 5,6% para estudantes de medicina e 12,7% para os demais cursos, enquanto a depressão moderada foi de 10,8% entre estudantes de medicina e 17,7% nos demais cursos^24^. O estudo de Leppink et al.^25^, realizado nos Estados Unidos utilizando o PHQ-9, encontrou a prevalência de 37,7% para sintomas depressivos leves a moderados e de 4,4% para sintomas depressivos graves, também inferior ao encontrado neste estudo.

O risco 50% maior de EDM entre as mulheres é consistente com a literatura, que aponta uma grande disparidade de saúde entre os gêneros, atingindo um pico na adolescência^4,5,13,14,26,27^. A diferença entre gêneros pode estar diretamente relacionada à vulnerabilidade, preconceito e discriminação de gênero e ao fato de as mulheres relatarem e reconhecerem melhor os sintomas depressivos e buscarem mais ajuda para problemas de saúde que os homens, bem como ao fato de muitas vezes enfrentarem jornadas múltiplas, cuidando da família, do trabalho e do estudo^28^.

A maior prevalência de EDM entre os universitários foi encontrada na faixa de 21 a 23 anos, semelhante à observada por outros autores que detectaram a maior prevalência de depressão entre os estudantes mais velhos, com 20 anos ou mais^5,8^. Olsson e Von Knorring^29^ observaram que 25% dos adultos com transtornos depressivos relatam ter tido seu primeiro episódio depressivo antes dos 18 anos.

A associação positiva entre a presença de histórico familiar de depressão e EDM está de acordo com a literatura^30,31^. Enquanto alguns estudos afirmam que a relação entre histórico familiar e depressão é genética, outros consideram que está mais relacionada ao convívio familiar^32^. Bahls et al.^33^ apontaram em sua revisão sistemática que o histórico familiar de depressão é o principal fator de risco para seu desenvolvimento entre crianças e adolescentes, aumentando-o em até três vezes. A homossexualidade e a bissexualidade, orientação sexual de minorias, foram fatores de risco para EDM neste estudo, concordando com os achados da literatura^25,34^. A maior ocorrência de depressão na população que apresenta orientação sexual minoritária pode estar relacionada ao preconceito, falta de aceitação e estigmatização imposta pela sociedade, assim como ao distanciamento familiar e menor rede de apoio^34^.

O presente estudo encontrou a maior prevalência de EDM entre os estudantes que relataram morar com amigos ou colegas, o que pode estar relacionado à distância das suas famílias e rede de apoio, bem como à necessidade de adaptar-se à rotina de repúblicas, pensões e moradias compartilhadas^7,23^.

Apesar de a maioria dos estudos encontrados na literatura tratarem especificamente de universitários dos cursos da área da saúde, o presente estudo observou as maiores prevalências de EDM entre estudantes das áreas de humanidades, artes e linguística, resultado semelhante a outro estudo brasileiro^14^. A maior prevalência de depressão em estudantes da área de humanidades pode ter relação com cobranças e rotinas acadêmicas particulares e/ou maior dificuldade dos estudantes destas áreas para enfrentá-las^14^. Estudos anteriores observaram que a insatisfação dos estudantes com o curso também esteve positivamente relacionada com a ocorrência de EDM e TDM^27,30^.

Foi observada uma relação negativa entre desempenho acadêmico e o EDM, assim como já descrito na literatura. O bom desempenho acadêmico é um marcador significativo de sucesso e desenvolvimento dos estudantes, indicando uma capacidade de resposta positiva à competitividade e ao alto nível de exigência existente na universidade. Essa associação está sujeita à bidirecionalidade, uma vez que o estudante já deprimido tende a encontrar maiores dificuldades na realização de suas atividades diárias, assim como pode ficar deprimido pelo baixo desempenho alcançado. Além disso, estudantes com EDM podem fazer uma autoavaliação mais crítica e pessimista^4,7,12,27^.

A associação positiva entre o consumo de drogas ilícitas, álcool e depressão é consistente com os achados da literatura^23,26^. A relação causal entre esses desfechos não está totalmente elucidada, sendo descrito que alterações neurofisiológicas e metabólicas resultantes da exposição ao álcool podem estar relacionadas ao desenvolvimento da depressão^35^. O consumo de drogas pode estar relacionado ao EDM tanto como fator de risco para o desenvolvimento de alterações bioquímicas e neurofisiológicas, bem como por ser um marcador de vulnerabilidade do universitário. A literatura relata que o consumo de drogas ilícitas está relacionado a pensamentos suicidas, tristeza, solidão e dificuldade para dormir, e que a presença do uso abusivo de álcool dobra a chance da ocorrência do transtorno depressivo, sendo o contrário também verdadeiro^35,36^. Assim, considerando o delineamento transversal, essa associação pode ser afetada por causalidade reversa e/ou bidirecionalidade.

O presente estudo não encontrou associação entre o nível econômico e a cor da pele com EDM, embora essas associações sejam amplamente relatadas pela literatura. A política de cotas existente na instituição busca ampliar o número de estudantes de escolas públicas, negros e índios. Embora os estudantes cotistas provenham de grupos mais vulneráveis, o árduo processo seletivo faz com que ingressem na universidade os mais privilegiados dentre seus pares, aqueles mais proativos ou com melhor capacidade para enfrentar as adversidades, o que pode explicar a ausência de associação com EDM^4,5,31^.

A mudança de semestre letivo no período de coleta de dados e a desistência ou abandono do curso por parte dos estudantes implicou uma perda considerável, mais concentrada em homens e em indivíduos mais velhos, de cursos das ciências exatas e da terra/agrárias e engenharias, o que pode provocar imprecisões na estimativa de prevalência. Embora o estudo avance em relação à literatura ao avaliar a totalidade dos cursos, a validade externa dos achados é limitada por referir-se a uma universidade específica.

Como aspectos positivos destaca-se o alto valor descritivo e de rastreamento para a saúde mental dos estudantes, que servirá de base para acolhimento e intervenções a serem realizadas na população universitária, e o fato de o instrumento ter sido autoaplicado, possibilitando ao estudante liberdade para abordar assuntos delicados como o EDM e seus sintomas. O fato de o PHQ-9 não ter sido utilizado em outros estudos em universitários brasileiros limita a comparabilidade dos achados; no entanto, como ele foi validado para a população brasileira recentemente e tem a vantagem de fornecer diagnóstico de EDM, é desejável que futuros estudos passem a utilizar esse instrumento^17^.

Conclui-se que, além dos aspectos individuais, familiares e comportamentais, semelhantes aos já descritos como fatores de risco para EDM na população em geral, aspectos acadêmicos também influenciam a ocorrência de depressão entre universitários. Considerando a alta prevalência de EDM e seu impacto negativo na saúde, na qualidade de vida e no desenvolvimento acadêmico dos universitários, são necessárias políticas públicas e institucionais que enfoquem a promoção da saúde, em especial da saúde mental, bem como estrutura para atenção adequada a essa demanda dos estudantes.
